# Smart Porous Multi-Stimulus Polysaccharide-Based Biomaterials for Tissue Engineering

**DOI:** 10.3390/molecules25225286

**Published:** 2020-11-13

**Authors:** Fernando Alvarado-Hidalgo, Karla Ramírez-Sánchez, Ricardo Starbird-Perez

**Affiliations:** 1 Centro de Investigación en Servicios Químicos y Microbiológicos, CEQIATEC, Escuela de Química, Instituto Tecnológico de Costa Rica, Cartago 159-7050, Costa Rica; 2 Master Program in Medical Devices Engineering, Instituto Tecnológico de Costa Rica, Cartago 159-7050, Costa Rica; 3 Centro de Investigación en Enfermedades Tropicales, CIET, Facultad de Microbiología, Universidad de Costa Rica, San José 11501-2060, Costa Rica

**Keywords:** biomaterials, porous materials, biomimetic, multi-stimulation, tissue engineering, conductive polymers

## Abstract

Recently, tissue engineering and regenerative medicine studies have evaluated smart biomaterials as implantable scaffolds and their interaction with cells for biomedical applications. Porous materials have been used in tissue engineering as synthetic extracellular matrices, promoting the attachment and migration of host cells to induce the in vitro regeneration of different tissues. Biomimetic 3D scaffold systems allow control over biophysical and biochemical cues, modulating the extracellular environment through mechanical, electrical, and biochemical stimulation of cells, driving their molecular reprogramming. In this review, first we outline the main advantages of using polysaccharides as raw materials for porous scaffolds, as well as the most common processing pathways to obtain the adequate textural properties, allowing the integration and attachment of cells. The second approach focuses on the tunable characteristics of the synthetic matrix, emphasizing the effect of their mechanical properties and the modification with conducting polymers in the cell response. The use and influence of polysaccharide-based porous materials as drug delivery systems for biochemical stimulation of cells is also described. Overall, engineered biomaterials are proposed as an effective strategy to improve in vitro tissue regeneration and future research directions of modified polysaccharide-based materials in the biomedical field are suggested.

## 1. Introduction

The number of publications related to the tissue engineering field has increased dramatically in recent years, referring to the potential regenerative methods and strategies for almost every tissue and organ of the human body. Progress has been reached by the integration of interdisciplinary research from cell biology, biomaterial sciences, and medical fields [1]. Specifically, tissue engineering involves the design and synthesis of three-dimensional (3D) matrices from biomaterials to provide a structural framework and to facilitate the attachment and migration of host cells, inducing a successful in vitro and *in vivo* regeneration of tissues [2,3,4]. Biomimetic 3D scaffolds may allow the control and application of a multi-stimulus to cells, including mechanical, electrical, and biochemical stimulations, in order to trigger specific responses, such as cell differentiation and tissue repair [5,6,7,8].

Tissue regeneration is naturally mediated by molecular processes, which direct gene expression to control renewal, restoration, and cell proliferation [9]. Nevertheless, normal regeneration is affected by aging, diseases, or accidents [10,11]. Thus, the increasing incidence of skin, muscle, and bone disorders, suffered by many people around the world, has prompted a critical need to develop engineered strategies to improve the replacement and regeneration of biological materials [11,12,13]. While many repair techniques have been proposed over recent decades, most of the surgical interventions have been directed toward the treatment of clinical symptoms but none have successfully repaired damaged tissues [14]. Consequently, in recent years, tissue engineering and regenerative medicine studies are focused on using the regenerative abilities of cells, in combination with engineered biomaterials, to create implantable scaffolds for tissue regeneration and reparation [1,10]. 

Porous materials from polysaccharides have been used as extracellular matrices (ECM) in tissue engineering in order to generate diverse types of cell lineages, promoting regeneration [15,16], for instance, in stem cells [17], osteoblasts [18], skeletal muscle cells [19], and endothelial cells [20]. In the biomedical field, aerogels from different sources have found applications as implantable devices, dressings for wound healing, synthetic bone grafts, carriers for different drugs, biosensing, and biomedical imaging [6,21]. 

Since they were first fabricated in 1932, aerogels have become the subject of great interest for different application fields [22]. Most common aerogel sources are from inorganic or petrochemical-based materials, such as those used to produce silica and graphene aerogels [23,24]. Recently, large efforts have been dedicated to produce aerogels using polysaccharides as raw materials. Relating them with inorganic starting materials and those derived from fossil oil, natural polysaccharides are more sustainable, green, non-toxic [25], biodegradable [26] and they have more abundant natural sources [27]. Several examples of engineering porous materials from polysaccharides have been developed. Starch and alginate aerogels [28,29], starch microspheres [30], and cellulose nanowhiskers [31] are among the different examples found in the literature. From a basic science perspective, the capacity to modulate the biomaterial properties to convey unique material characteristics allows their application in different fields, with biomedical being the most important, from our point of view. 

Numerous strategies have been reported to obtain polysaccharide-based aerogels to guide functional restoration to the site of injury. Control of the size and porosity in the scaffold mediates cellular infiltration [32] and facilitates the transport of nutrients [33], oxygen [34], and waste products [35]. Porosity also regulates the vascularization by angiogenesis and cell attachment [30,36]. Mechanical properties of biomaterials, such as stiffness, structure, and topography, are also considered during ECM synthesis, mainly because they can alter the local tissue microenvironments through intracellular and intercellular signaling [7,9,37]. Besides, one of the most relevant applications of polysaccharide-based aerogels is the capability of releasing drugs as controlled delivery systems. The synthetic scaffold acts as a carrier for drug molecules, in order to release them specifically to target cells or tissues and improve their differentiation and regeneration [6,38,39]. Specifically, the combination of polysaccharide-based porous materials with biomolecules is known as a polymer bioconjugate and is a novel strategy used for the fixation of amino acids, nuclei acids, peptides, and carbohydrates to different polymers, in order to improve their application as therapeutics [40]. Alternatively, conductive polymers have been proposed in combination with aerogels as a system for electrical stimulation of cells and tissues in regenerative medicine [5,41].

Our review summarizes the current status of smart 3D scaffold systems based on polysaccharides regarding their production, properties, and potential applications in the biomedical field. Although those topics have been extensively reviewed in the past, our approach will focus on the potential development of biomimetic 3D scaffold systems including the physical, mechanical, electrical, and biochemical properties of modified polysaccharide-based aerogels and cryogels. Moreover, novel research directions of these smart materials, including strategies for the impregnation of drugs and their subsequent release from porous materials, and modification with conductive polymers were covered to be applied in the biomedical field.

## 2. Overview: Polysaccharide-Based Porous Materials 

Aerogels are solid, lightweight, and high specific surface area materials with interconnected networks of particles obtained from a wet gel during a process where their liquid phase is removed and replaced with gas without the collapsing of the solid structure [6,22]. 

Through time, aerogels have been obtained by structuring both organic and inorganic materials. Silica [24,42,43], silica/pre-polymerized vinyl trimethoxy silane (VTMS) composites [44], and graphene-based aerogels [23,45] are among the most used inorganic materials reported for aerogel production. However, despite several relevant features found for inorganic aerogels, biopolymer-based aerogels have been the object of much research lately due to their mechanical properties [46], non-toxicity [25], and biocompatibility [21], all desirable properties in systems to be used in biomedical field [47]. 

Polysaccharide-based aerogels were reported first by Kistler [22], using cellulose, nitrocellulose, gelatin, agar, and egg albumin. More recent research has reported the obtention of aerogels from polysaccharides such as chitosan [48], chitosan/alginate [49], cellulose [50], starch [13,30,51,52,53], starch/κ-carrageenan (κC) [53], and pectin [27]. 

Considering that polysaccharides possess abundant natural sources from which they can be obtained [27], along with renewability and non-toxicity, they are excellent raw material candidates for aerogel processing regarding circular economy principles, relying on renewable raw material or energy sources [25,54]. 

### 2.1. Processing Strategies for Polysaccharide-Based Aerogels

**Figure 1:**
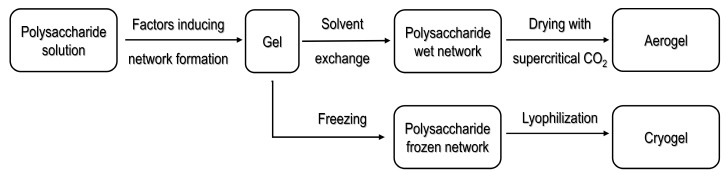
Pathway for porous materials produced by supercritical drying as well as freeze drying. Modified from [55] under Creative Commons attribution license.

Diverse strategies have been used to obtain polysaccharide-based aerogels. The sol-gel method is commonly reported as an initial step in the processing pathways for organic or inorganic materials [28]. In the sol-gel process, a hydrogel formation is induced by crosslinking of the base material. Once the hydrogel is formed, it is necessary to select the drying method to be used; materials obtained from supercritical drying are commonly known as aerogels, whereas materials dried by freeze drying (lyophilization) are known as cryogels [21]. Figure 1 illustrates the scheme for supercritical drying and freeze drying, the most widely used methods in processing porous materials [21]. 

#### 2.1.1. Processing Using Supercritical Fluid Technology

The usage of supercritical fluid technology allows the material design with different composition, morphology, porosity, and linear architecture [56]. In addition, processing with supercritical fluids (SCFs) leads to a solvent-free end-product with high purity. This environmentally friendly feature has been noted by other studies. In fact, SCFs have been regarded as “the green solvents for the future” since they compress different ecological benefits, an emphasis is made on their low energy consumption [21,57]. 

Carbon dioxide (CO_2_) is the most widely used supercritical fluid, in part due to the mild operating conditions, 7.38 MPa and 304 K [56]. Supercritical drying (SCD) avoids the formation of the vapor–liquid interface that occurs upon solvent evaporation. When evaporation of the solvent occurs, the capillary pressure gradient on the pore walls may reach up to 100–200 MPa [28]. Aerogels processed by means of SCD tend to show a mesoporous structure (pores of a 2–50 nm diameter) and thus require a templating technique for inducing the formation of macropores [56]. Finally, SCF technology has been applied not only to obtain porous materials but also as a strategy for the sterilization of polymeric scaffolds from aerogels [13]. 

#### 2.1.2. Cryogels Obtained by Freeze Drying

Freeze drying (FD), or lyophilization, is a drying process in which the solvent or the medium of suspension is crystallized at low temperatures and is thereafter sublimated from the solid state directly into the vapor state [58]. It is reported as a simple, environmentally friendly and economic technique for producing highly porous cryogels with reduced shrinkage [21]. 

Significant advantages of using FD during cryogel synthesis are that the whole conversion of raw materials and the recycling of water without pollution or volatile organic compounds problems are achieved [59]. High safety derived from its straightforward operation is an important feature that has been remarked in the literature [27]. Nevertheless, one important drawback reported for freeze drying is that the process takes several hours to be completed [23]. In addition, freeze-dried materials tend to have larger macroporosity (pores >50 nm diameter) than SCD-processed materials [21]. 

Freeze drying requires freezing the hydrogels, transforming all the liquid that fills the interconnected 3D structure, to solid. Then, at low pressures, the sublimation of the solid solvent is promoted, avoiding the formation of the vapor–liquid interface [51,59]. The morphology of the porous structure is determined by the nucleation and ice crystal growth process of the gel solution [27], producing cryogel pores due to sublimation of the ice crystals [60]. Large ice crystals are obtained with low nucleation rates; this is reached by using small subcooling temperatures, as close to the equilibrium state as possible, between solution and ice crystals (0 °C) [58]. 

Two important steps are found for the crystallization process: nucleation and ice crystal growth [27]. Since pores are formed due to the sublimation of the ice crystals [60], the crystal morphology has a direct effect on the final pore morphology of the cryogel. The crystal morphology can be related to the freezing or and pre-freezing conditions (temperature and rate), additives, suspended solids [60], or the initial material concentration [27]. In addition, increasing the pressure at the freezing phase can shorten the cooling time and form small regular ice crystals [27]. 

Direct comparison between the porous materials obtained by SCD or by FD results in an important specific surface area decrement for the freeze-dried cryogels [51]. However, cryogels have shown porosity values equal to or higher than SCD aerogels, with an important macropore fraction that may be suitable for different applications where macroporosity is required. See detailed information in Table 1. 

## 3. Polysaccharide-Based Porous Materials for Tissue Engineering

In recent years, tissue engineering and regenerative medicine studies have been based on the combination of specific types of cells and 3D porous scaffolds to induce a successful in vitro regeneration of diverse tissues [2,3,4].

The main efforts on engineered ECM in the biomedical field have been focused on the use and stimulation of pluripotent stem cells, which are special cells that have the ability to perpetuate themselves through a mechanism of self-renewal and to generate diverse types of cells through differentiation processes [15,16,17]. Nevertheless, osteoblasts [18], skeletal muscle cells [18], and endothelial cells [20] have been also studied.

### 3.1. Polysaccharide-Based Porous Materials as Extracellular Matrices

An extracellular matrix is an organized network composed by a mixture of cellular and non-cellular components. It plays an important role in tissue and organ morphogenesis, cell function, and structure maintenance. The biochemical and mechanical stimulus that cells receive from the matrix influences their growth, migration, differentiation, survival, and homeostasis [73].

Aerogels, as porous 3D matrices, possess a nanostructure that is able to mimic the extracellular matrix of the natural tissue, providing a favorable environment for the regeneration of tissues and organs [6,74]. Coupled with high porosity, low densities, and high inner surface areas, porous materials can provide appropriate morphology engineering, opening the possibility for their application as synthetic scaffolds for tissue engineering [52].

A scaffold acts as a template for new tissue formation [75] and its 3D structure guides the proliferation and colonization of cells, promoting tissue growth [56]. An ideal synthetic ECM should exhibit a highly open and uniform porosity, over 80%, with micro- and mesopores that enable cell attachment and macropores for proper vascularization [56]. The configuration of the scaffold topology is critical in controlling cellular function, it should match the endogenous topology of the cell membrane in order to enhance signaling and function [36].

Nowadays, regenerative medicine is focused on the evaluation of novel skeletal muscle regeneration strategies, which involve the prefabrication of muscle tissues in vitro by differentiation and maturing of muscle precursor cells on a scaffold, providing the required environment for myogenic differentiation of the cultured cells [76]. Researchers are studying the incorporation of products obtained from cellular metabolism in synthetic ECMs. These materials are mainly constituted of glycosaminoglycans, a group of polysaccharides that can modulate cell activity by mimicking aspects of the *in vivo* extracellular environment, providing important roles in cell signaling, proliferation, and differentiation through their ability to interact with ECM proteins and growth factors [77,78,79,80,81]. Hyaluronic acid, heparan sulfate, and heparin are the most used glycosaminoglycans in synthetic ECMs, mainly to direct the differentiation on mesenchymal stem cells (MSC) [82,83].

The synthesis of alginate hydrogels for platelet-rich plasma encapsulation as a coating for polylactic acid porous devices is another strategy used to improve cellular responses on synthetic ECM, the hydrogel system allows for better cellular integration and influences the vascularization into the membrane after skin implantation of the device, and the access to nutrients and growth factors was also improved with the engineered hydrogel. Platelet-rich plasma hydrogels could also support oxygenation of cells, avoiding hypoxia immediately post-transplantation [84,85]. In a similar study, calcium peroxide (CPO) was used during the synthesis of a gelatin methacryloyl bioprinted scaffold to achieve improved cellular oxygenation and increase fibroblast viability under hypoxia conditions [86].

**Figure 2:**
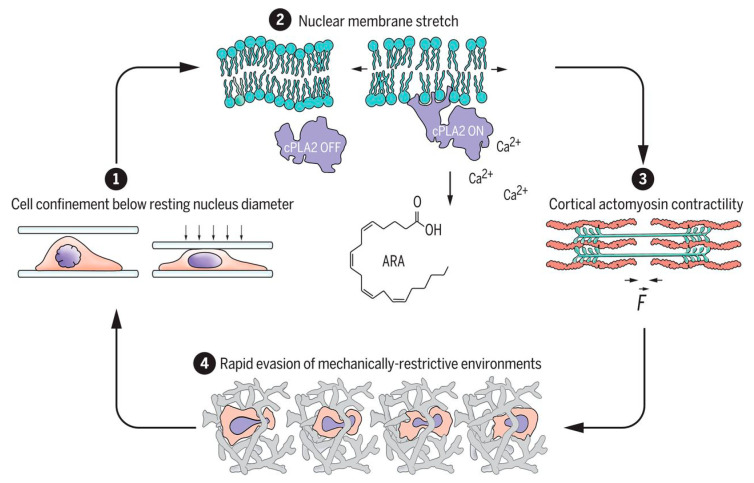
Schematic representation of nuclear deformation and stretching of the nuclear envelope after cell compression (1), which cause calcium release, phospholipase A_2_ activation, and arachidonic acid production (2), for the regulation of actomyosin (3) and the increasing of cell migratory capacity through the 3D matrix (4). Reproduced from [89] under Science Copyright Clearance Center (CCC) license.

The spatial arrangement, porosity, biocompatibility, and proper scale of the ECM are some of the most important features that must be adjusted for use in nervous tissue, skin, bone, and muscle [76]. Nevertheless, several other factors, such as mechanical properties and chemical modification of scaffolds, significantly influence cellular behavior [5,87]. For example, recent studies have shown that the cell nucleus works as a fast mechanical respondent in cell contractility events because of the three-dimensional extracellular matrix restriction environment, inducing deformation and the movement of cells through the activation of cytosolic phospholipase A_2_ and arachidonic acid, which regulate myosin activity [88,89] (Figure 2).

### 3.2. Influence of the Mechanical Properties of the Scaffold in Cells and Tissues Behavior

The main goal of tissue engineering and regenerative medicine is to create strategies for replacing defective tissue. The use of polymeric scaffolds as extracellular matrices tries to mimic the *in vivo* host conditions to restore or improve the regeneration of damaged tissues. An extracellular matrix requires not only pore size control to induce cell adhesion and the ingress of nutrients and oxygen but also the incorporation of signal molecules, such as growth and differentiation factors, as well as a proper matrix architecture and mechanical properties to keep the implanted cells alive [46,90,91,92,93,94].

The mechanical characteristics of a scaffold for in vitro or *in vivo* cell studies may ultimately impact how the hosted tissue responds to the scaffold [87]. In this regard, the architecture, chemistry, topography, and physical properties of the employed scaffold as an ECM influence the structure and function of the surrounding tissue. Cells are constantly subjected to physical forces from their microenvironment. Mechanical properties of the porous materials are indispensable to determine the viability of a tissue and play a crucial role in cellular phenotype and homeostasis [95]. There are several types of cells that respond to a mechanical stimulus. The mechanoresponsive cells include chondrocytes [88], cardiomyocytes [94], osteoblasts [96], muscle cells [97], endothelial cells [98], stem cells [7], and other tissue connective cells.

Atomic force microscopy (AFM) analysis, magnetic resonance elastography (MRE), shear rheometry, micropipette aspiration, and microindentation are some techniques commonly used to determine systematic cell responses, induced by mechanical properties of scaffold [95]. These methods cause the compression, bending, twisting, and stretching of the scaffold [99,100,101], inducing specific cellular responses.

Cells may sense physical cues, such as osmotic pressure, shear force, and compression loading, as well as architecture, rigidity, and other several properties of the ECM, through a process known as mechanotransduction [95]. Thus, mechanotransduction corresponds to the cell capacity to transform a mechanical stimulus into biochemical signals. There are surface proteins in cell membranes which detect a force differential and then amplify and propagate this mechanical signal to elicit a change in cell behavior [37,95].

Compression and shear stress, caused by the synthetic ECM in a cell culture, transfer mechanical stimulation to the cells and enhance their biochemical signaling. The upregulation of gene expression and the changes in cellular metabolism during mechanical stimulation are regulated by mechanically sensitive surface receptors on cell membranes. There are several proteins related with the mechanotransduction to biochemical events, integrins, specifically β1 and α5β1 integrins, are the best proteins studied so far [37].

Scaffold stiffness has been shown to have a significant impact on numerous cells and their fate, such as cell adhesion, cytoskeleton rearrangement, cell migration, stem cell differentiation, and muscle cell contractility [87]. The stiffness of an ECM in 2D cell cultures may influence the differentiation pattern of a same cell type; it has been reported that a soft matrix (0.1–1 kPa) promotes neurogenic differentiation, matrices with a medium stiffness (8–17 kPa) promote myogenic differentiation, and matrices with high stiffness (25–40 kPa) promote the osteogenic differentiation of mesenchymal stem cells [9]. Several authors have reported that the stiffness of a synthetic ECM induces mechanical stimulation of cells and the subsequent expression of cellular differentiation markers [93], tissue organization [97], causes the synthesis of extracellular matrix components [93], changes cell morphology, and improves their adhesion to synthetic scaffolds [93,98]. Additionally, the positive inotropic and chronotropic responses to both ion concentration (i.e., calcium, Ca^2+^) and temperature after mechanical stimulation of cardiomyocytes are also reported [94].

Complexity of the mechanotransduction induced by integrins is multifaceted as the proteins can form 24 possible functional distinct dimers and each dimer forms diverse complexes with multiple intracellular adaptor proteins to dictate the interplay between biochemical and cytoskeletal elements to determine their contribution to cellular mechanoresponses [37,93]. Nevertheless, it is well known that efficient force transfer and associated cytoskeleton changes are correlated with focal adhesion formation, as defined by the recruitment of talin, vinculin, and α-actinin to the stimulated integrin; these focal adhesion proteins form the molecular bridge that physically interlinks integrins with actin microfilaments [9,92,102].

Cytoskeletal changes caused by mechanical stimulation of cells are influenced by several biochemical pathways. It has been reported that maturation of focal adhesions causes activation of focal adhesion kinase (FAK); the scaffold protein, associated with adhesion plaque, triggered the Rho-associated protein kinase cascade (ROCK), which enhanced cellular tension through engagement of actomyosin contractility [95,103]. ROCK protein involves several downstream signals, including extracellular signal-regulated kinases (ERKs) and the hippo pathway, which is related with yes-associated protein 1 (YAP1); both biochemical pathways translocate some activated proteins to the nucleus and associated with transcriptional factors to regulate cell proliferation, tissue growth, and differentiation, as well as cell migration [89,104,105]. The chronical cellular tension reinforces these downstream signaling pathways to potentiate the production of ECM and ECM remodeling proteins that stiffen the local microenvironment and reinforce mechanosignaling [95].

Experiments related with the mechanical stimulation of cells have been carried out since 1938, when Glücksmann studied endosteal cells from embryonic chick tibiae [106]. Cells were grown on substrates of explanted intercostal muscle, to which pairs of neighboring ribs were left attached [106]. After several days, cells were compressed when the ribs were drawn near toward one another as the muscle tissue degenerated. Table 2 summarizes the main strategies to induce mechanical stimulation of synthetic ECM and their effect in cultured cells.

The study of mechanical properties of extracellular matrices is important to ensure resistance of cultivated cells to *in vivo* stress were the matrix is used to replace damaged tissue [110,111]. Cellular responses depend on the magnitude and duration of the stimulus and high pressures may cause damage to the cell membrane and nucleus, followed by inflammatory reactions due to tissue breakdown *in vivo* [101]. Additionally, stiffness, roughness, and viscoelasticity are important in directing the immune response of cells. There are several T cell receptors that act as mechanical sensors, enabling the T cells to discriminate between a wide range of stiffness found in the body and respond accordingly [9]. Thus, hydrogels with higher stiffness stimulate the production of both pro- and anti-inflammatory cytokines, in contrast with low stiffness hydrogels, where the inflammatory response is suppressed and results in an overall lower foreign-body reaction *in vivo* [9]. The effect of the substrate mechanical properties on the in vitro response of macrophages has been also studied using poly(ethylene glycol) hydrogels (PEG) [87]. Results showed that stiffness did not impact the macrophage attachment; nevertheless, it elicited differences in their morphology.

The mechanical characteristics of scaffolds can be adjusted using adequate dynamic biomaterials in order to create matrices with an appropriate stiffness to direct specific cellular responses. Mechanical properties of synthetic scaffolds are also used to design stimulation protocols to induce the controlled release of responsive drugs potentially used for tissue regeneration.

### 3.3. Polysaccharide-Based Porous Materials as Scaffolds for Electrical Stimulation of Cells

Another research field of interest is focused on the preparation of electrical systems to induce specific cellular responses. Diverse tissues (e.g., nerve, muscle, and glandular) make use of endogenous electric fields (EF) to transmit electrical signals. The endogenously-generated EF exists in both the cytoplasm and extracellular space [112]. Ionic currents and EFs in living cells play critical roles in important biological processes as they generate electromotive force, maintain a required electric potential, and allow some cellular functions [113,114]. These bioelectric signals are generated by gap junctional connections and ion channels or pumps moving ions, mainly potassium (K^+^) and chloride (Cl^−^), across the membrane [113,115], and the regulation in cellular physiology is induced by pH gradients, specific ion flows, and changes in transmembrane potential [116].

Currently, exogenous electrical stimulation of cells is a widely used method to improve their biological functions. Many authors have reported the use of nerve [117], bone [118], muscle [119], and neural stem cells [120], because their extensively recognized piezoelectric characteristics make them attractive for research on the role of exogenous electrical stimulation.

Coupling of an electromagnetic field with a live cell can occur via field interaction with charged molecules and proteins in the cell membrane [114]. The application of an electrical stimulus to induce cellular responses depends mainly on the level and nature of the electric potential or current applied, the frequency of the stimulus, and the type of cell studied [113]. It is reported that the application of the EF in a culture medium affects the migration [121], orientation [122], proliferation, and differentiation of cells [123,124]. Nevertheless, in most cases, it is used specifically to revive damaged or disabled tissues in the neuromuscular system as well as to accelerate the healing of injured musculoskeletal tissues, such as bone, ligament, and articular cartilage [112].

In this regard, biomaterials may receive considerable attention for their influence on cellular behaviors, ability to mimic biological functions, and, more recently, as electronic conductive systems with a potential use as tissue engineering scaffolds [5,125].

**Figure 3:**
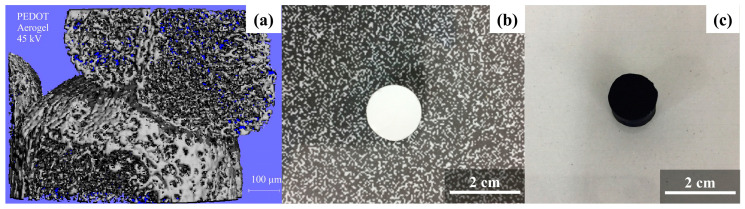
Porous material microtomography (micro-CT) image (**a**) and aerogel images before (**b**) and after conductive polymer (i.e., poly(3,4-ethylenedioxythiophene) (PEDOT)) modification (**c**). Reproduced from [52,53] under Elsevier Copyright Clearance Center (CCC) licenses.

Some electroactive materials, such as conductive polymers (CPs) (e.g., poly(3,4-ethylenedioxythiophene) (PEDOT)), which are a special class of polymeric materials that present electric and ionic conductivity, are currently being studied in combination with aerogels or cryogels as a promising field in regenerative medicine (Figure 3). Nevertheless, in the past, research studies have extensively used this kind of polymer to create organic conductive interfaces, neuroprosthetic devices, neural probes, and controlled drug-delivery systems [5,41,126].

Conductive polymers can be structured with porous systems using different techniques [127,128,129]. Starch and starch/κ-carrageenan aerogels have been used as templates for the obtention of nanoporous conductive materials [52,53]. In the biomedical field, conductive nanoporous materials have been applied not only as physical support but also as a medium to provide electrical stimulation of a cell culture. Electrical stimulation in neural cells has shown great potential for function restoring and wound healing [130].

On the other hand, the incorporation of anionic drugs and κ-carrageenan on the structure of starch porous materials is particularly interesting since both compounds may act as dopant agents for the conductive matrix, as it was shown recently [39,53,131]. Dexamethasone, a well-known glucocorticoid anionic drug, has recently been the object of research from an electrochemical point of view, regarding its doping properties on conductive matrices [132] and for its ability to be released by electrochemical stimulation from a PEDOT/κ-carrageenan film [39]. The above opens the possibility to create scaffolds from conductive porous materials and the incorporation of specific drugs in their structure to be applied as stimulation systems in tissue engineering.

### 3.4. Polysaccharide-Based Porous Materials as Drug-Delivery Systems

One of the main approaches and most relevant applications of biopolymer-based aerogels is their use as drug-delivery systems [6,38]. The application of these materials as controlled drug-release matrices has gained interest in the last years due to aerogel properties, such as its high surface area, high porosity, and biocompatibility [38]. Aerogels can act as a carrier for bioactive compounds, showing high loading capacity, enhanced stability upon storage, and accelerated drug release, if required [48]. Along with the high loading capacity, biopolymer-based aerogels also show an improved dissolution rate of poorly water-soluble drugs [6].

The biocompatibility of natural polymers along with the outstanding performance of aerogels as carriers for active compounds, such as drugs, have promoted the systems as scaffolds in body implants to accelerate tissue formation by providing a suitable porous structure that promotes cell colonization [62,133]. Diverse authors have also studied the incorporation of drugs and growth factors to promote the attachment, proliferation, and differentiation of cells, in order to provide both substitutes for damaged tissues and therapeutic schemes that reduce post-implantation inflammation and infections [12,133,134,135].

Controllable drug-release systems may be categorized as mechanical methods, which are mainly *in vivo* implantable pump delivery systems built from biocompatible nanomaterials [136,137], and as polymeric drug delivery systems. The last one makes use of biopolymers, in which the delivery of drugs is mainly dominated via diffusion and recently by electrochemical methods [137]. Hence, the incorporation of drugs within these kinds of porous scaffolds has been studied previously for osteogenic differentiation, bone repair activity, and the stimulation of neural tissues [126,133].

#### 3.4.1. Diffusive Phenomena on the Controlled Release of Drugs on Polysaccharide-Based Aerogels

Different methods for drug impregnation or loading can be found in literature regarding porous materials from polysaccharides. Supercritical technology employing scCO_2_ has been defined as the most innovative technique for producing polymer/drug composite systems for pharmaceutical applications [138]. By means of supercritical fluid technology, the impregnation of aerogel particles with drugs such as ketoprofen was achieved [62]. This process consists of placing aerogel particles and ketoprofen in a closed autoclave under agitation; the ketoprofen was dissolved in scCO_2_ and adsorbed in the aerogel matrix [62]. The same procedure was reproduced for obtaining poly(ɛ-Caprolactone) (PCL) scaffolds loaded with ketoprofen [72] and for alginate-based aerogel microparticles for mucosal drug-delivery [38]. In addition, maize starch aerogels and calcium alginate aerogels were impregnated with different non-steroidal anti-inflammatory drugs, such as nimesulide, ketoprofen, and diclefenac sodium [138].

Supercritical CO_2_ was also applied in the impregnation of starch and sodium alginate aerogels with five different active compounds, namely loratadine, ibuprofen, rifabutin, dihydroquercetin, and artemisinin, showing enhanced releasing times as well as double bioavailability in some drug–aerogel systems [139]. In addition, this research concludes that the affinity between the aerogel and the active substance must be high so that the active compound loading will be high enough to provide an increase in the dissolution rate and bioavailability [139].

Another method for loading aerogel particles was reported by mixing the active compound (Vancomycin) with a chitosan solution in different weight ratios, thus obtaining vancomycin-loaded chitosan aerogel particles, which are proposed as a system for fast local administration of the antibiotic for wound dressings [48]. A similar procedure was performed by [67], where mesoporous starch aerogels were loaded with celecoxib by adsorption during the solvent exchange steps.

Three steps are considered for the diffusion model: first, the film diffusion; the second step is the slowest, thus controlling the kinetics of the phenomenon, and it is called intraparticle diffusion; finally, the last step is the adsorbate release on adsorbent active sites [140]. Several works have been published regarding the release of drugs by means of diffusion phenomena [38,48,62,71,72,139,141]. The first mechanism when a drug-loaded polymeric material meets an aqueous solution is the filling of the pores near the surface; then, drug diffusion is initiated by the dissolution of the solute in the water-filled pores and the continuous diffusion in water [142]. Through time, the polymeric network starts swelling, inducing several structural changes that are affected by the cross-linking density and the degree of crystallinity of the 3D network. From the swelling of the polymer, a new diffusion starts through the swelled polymer structure [142]. By analyzing the release profile of drugs, conclusions can be obtained on whether the kinetics follow a Fickian or non-Fickian diffusion profile [143,144].

Innovative drug delivery systems are not only studied to improve cellular responses in different tissues but as a strategy that develops platforms and nano-scale devices for selective delivery of therapeutic small drug molecules to the cells or tissues of interest, for the maintenance of appropriate doses, and to improve individual therapy. To meet this demand, many drugs have been reformulated in new drug delivery systems to provide enhanced efficiency and more beneficial therapies [136,137].

#### 3.4.2. Controlled Drug Release by Electrical Stimulation Employing Conductive Porous Materials

In order to prevent the negative effects resulting from exposure to high dosages of drugs, local electronically-controlled release of pharmaceutical compounds from implantable devices appears as a promising option [145]. Drugs anchored inside the conductive materials have been reported using supercritical technology and electropolymerization [126,146].

Electrochemical methods involve the use of conductive polymers, which are electrochemically oxidized during the polymerization processes, generating charge carriers, and, thus, allowing ionic drugs’ impregnation based on electrostatic interactions [147]. There are two main electrochemical methods to induce the immobilization of drugs. In the first one, an ionic drug (preferably anionic) acts as a doping agent and its anchoring proceeds simultaneously with the process of matrix formation, commonly named one-step immobilization or *in situ* immobilization [146,148,149]. Drug fixation is the result of the ion-exchange processes during polymer oxidation. Ionic drugs can serve as counter-ions for the positively charged centers in the growing polymer chain [149]. Anti-cancer drugs, anti-inflammatory compounds, and hormones have been fixed on conductive materials using one-step immobilization, mainly for the development of neural devices [150,151,152].

The second method corresponds to the two-step or *ex situ* immobilization. The incorporation of the drug is carried out after the synthesis of the matrix, through ion exchange processes taking place at their surface. First, the polymer film is synthesized from a solution consisting of the monomer and a small ionic molecule as doping agent, without the drug. The obtained film is later reduced and oxidized by an electrical stimulus [148,149]. Reduction induces the removal of the dopant from the film; meanwhile, the drug, which acts as the second doping agent, is incorporated during the process of matrix oxidation [149]. This approach allows to prevent the interference of drugs during the growth of polymer matrix and their subsequent release does not have much impact on their physicochemical properties [148,149].

Related with the above, some strategies of drug fixation on conductive polymers using two different doping agents have been reported [39,149,153]. The anti-inflammatory drugs dexamethasone and κ-carrageenan were anchored simultaneously during PEDOT film formation, using *in situ* immobilization. After film oxidation, κC was maintained on the matrix, granting the film greater stability and integrity even after drug release [39].

Drug delivery is caused by electrochemical stimulation of the conductive matrix, which induces the oxidation and/or reduction of the film. By applying a negative potential, the polymeric matrix is reduced and the cationic charge of the polymer backbone is neutralized, causing the release of the anionic drug by electrostatic mechanisms [148]. In a similar procedure, applying negative and positive cyclic potentials induces the reduction and oxidation of the polymeric film, respectively; meanwhile, the matrix experiments expansion and contraction, which force the release of the drug. Although cyclic stimulation allows a greater amount of drug release in comparison with other methods, some authors have reported that the application of the stimulus may cause delamination, cracks, and breakdowns of the matrix, mainly in one-step immobilization systems [126,154,155].

**Figure 4:**
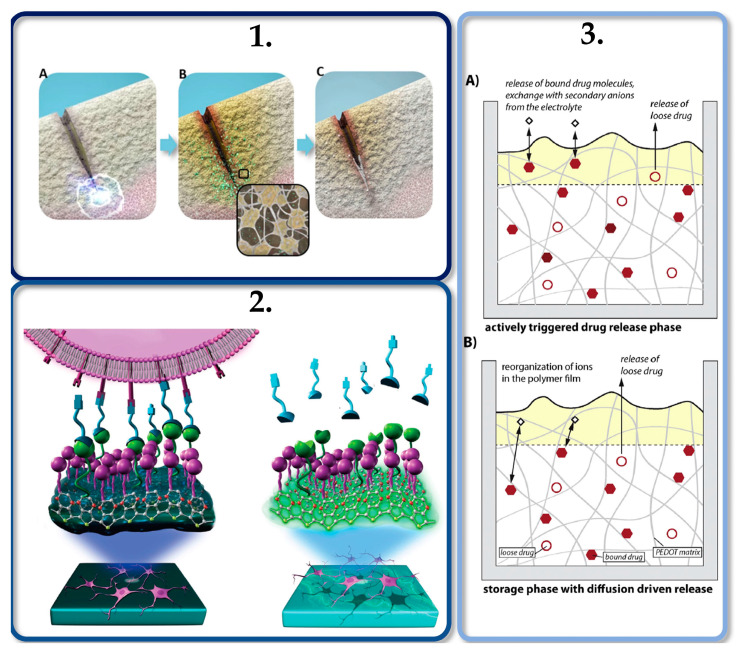
(**1**) Flexoelectricity induced by mechanical stimulation (**A**) plays an important role in bone repair and remodeling by inducing osteoblasts migration (**B**) and mineralization (**C**). Reproduced from [109] under Creative Commons Attribution License. (**2**) Electrical communication and redox-triggered interaction between neurons and (PEDOT) matrices functionalized with hydroquinone electroswitches and phosphorylcholine zwitterions. (**3**) Schematic representation of the active drug-delivery triggered by an electrical stimulus (**A**) and passive drug-release induced by diffusion processes from a conductive polymeric matrix (**B**). Reproduced from [151,158] under Elsevier Copyright Clearance Center (CCC) licenses.

The controlled release of drugs using electrical stimulation from conductive polymer films [39,126,151] opens the door for a different approach regarding the application of polysaccharide aerogels on drug delivery. Since these materials can be coated with an electrically conductive material while incorporating active compounds, those composites may be used in the controlled release of bioactive molecules by electrical stimulation [156,157]. These biochemical release systems are the main focus of several research groups and further investigations should follow this path in order to promote smart scaffolds that merge mechanical, electrical, and biochemical stimulation processes, mimicking the *in vivo* ECM conditions, in order to promote specific cell behavior, as shown in Figure 4.

## 4. Conclusions

The current status of biomimetic scaffold systems based on polysaccharides has been reviewed regarding multi-stimulation, mechanical, electrical, and biochemical, in order to trigger specific responses in cells during growth and differentiation, specifically in the biomedical field. Some details of their production and properties have been summarized, including modification with conductive polymers and strategies for controlled drug release from porous materials, such as aerogels. Therefore, future studies of modified polysaccharide-based aerogels for tissue engineering could consider promoting physical, mechanical, electrical, and biochemical multi-stimulation with the aim to mimic *in vivo* conditions.

## Figures and Tables

**Table 1:** Properties reported in different research studies for porous materials from biopolymers.

Raw Material	Fabrication Method	Specific Surface Area (m^2^/g)	Porosity (%)	Reference
Corn starch	scCO_2_	130–183	80–89	[13]
	scCO_2_	102–274	N.R.	[61]
	scCO_2_	221–234	85–90	[62]
	scCO_2_	79–87	N.R.	[52]
	scCO_2_	183–197	61–73	[51]
	FD	0.6–7.7	>80	[51]
	scCO_2_	223–247	87	[53]
	scCO_2_	313–362	N.R.	[63]
	scCO_2_	254	N.R.	[64]
	scCO_2_	370	N.R.	[65]
Wheat starch	scCO_2_	52.6–57.9	N.R.	[66]
	scCO_2_	34.7–60.9	91–93	[66]
Pea starch	scCO_2_	204–230	84–92	[62]
	scCO_2_	221	N.R.	[64]
Potato starch	scCO_2_	42–70	N.R.	[67]
	scCO_2_	85–88	N.R.	[64]
Starch/κ-carrageenan	scCO_2_	194–231	78–85	[53]
κ-carrageenan	scCO_2_	≈ 230	N.R.	[68]
Chitosan	scCO_2_	>250	>96	[48]
Cellulose	scCO_2_	287–303	92–96	[50]
	FD	297	96.4	[50]
	scCO_2_	20–246	91–99	[69]
Alginate/chitosan	scCO_2_	127.4–192.3	N.R.	[49]
Alginate composites	scCO_2_	200–800	N.R.	[70]
Whey protein isolate	scCO_2_	14–447	N.R.	[71]
	FD	<5	N.R.	[71]
Poly (ϵ-caprolactone)	scCO_2_	N.R.	54–58.8	[72]

N.R.: Not reported; scCO_2_: Supercritical CO_2_; FD: Freeze Drying.

**Table 2:** Used methods to induce mechanical stimulation of cells in synthetic extracellular matrices (ECM).

Raw Material	Mechanical Test	Result	Reference
Gelatin/nanohydro-xiapatite cryogels	Compressive mechanical stimulation of cryogels for 14 days in a bioreactor containing 150 mL of cultured medium at 30% compression strain.	Mesenchymal stem cells were attached to the scaffold and a higher extent of osteogenic differentiation was obtained after compression.	[7]
Self-assembled peptide hydrogel (arginine, leucine, aspartic acid, and alanine)	The hydrogel containing cells was placed into a hand-control stretch device for 120 h.	Smooth muscle cells resulting in a tight adhesion in the porous structure and a lineal cell proliferation rate were reported.	[46]
Poly(lactic-co-glycolic acid) fiber coated with polypyrrole	The electrical stimulation of the matrix induced their volume modification, causing changes in the mechanical strain.	The direct dual electrical and mechanical stimulation of the pluripotent stem cells cultured in the scaffold caused a faster expression of cardiomyocytes genes, important for myocardial regeneration.	[107]
Collagen matrix reinforced with rings of electrospun silk fi-broin mat	Dynamic stimulation with pulsatile or laminar flow. Pulsatile flow was induced with a gear pump which supply a steady flow (75 mL/min) in series with a pulsatile manifold. Laminar flow was carried out of steady flow of 75 mL/min.	Chondrogenic differentiation of MSCs was observed in the presence of chondrogenic supplements in laminar flow cultures. Pulsatile flow resulted in preferential cellular orientation, as dictated by dynamic circumferential strain, and induced MSC contractile phenotype expression.	[108]
Silicon tubes with inner surfaces modified with collagen type I solutions	Cells cultured on collagen-coated silicon tubes were exposed for 24 hours to the shear stress created when culture medium passes through the tube.	Mechanical stimulation caused by shear stress on adipose-derived mesenchymal stem cells depicted significantly higher gene expression of osteoblasts and adipogenic lineages. Moreover, mechanical stimulus induced endothelial differentiation after the addition of VEGF on cultured medium.	[98]
Microcracked hydroxyapatite substrates	Bending the top surface of the cracked substrate in a piezoelectric actuator using a force of 50 N at 5 Hz for 150 s.	Flexoelectricity caused by mechanical stimulation on a hydroxyapatite substrate induced apoptotic responses on osteoblasts and osteocytes. Apoptosis was followed by proliferation of the cells adjacent to the crack, better attachment on the substrate, and an increased expression of osteocytes markers.	[109]
